# Targeting confluent areas of slow conduction and electrogram fragmentation for atrioventricular node re-entrant tachycardia ablation

**DOI:** 10.1093/europace/euae094

**Published:** 2024-04-12

**Authors:** Jesús Jiménez-López, Victor Bazan, Carlos E Gonzalez-Matos, Andrea Di Marco, Nicola Bottoni, Antonella Battista, Sebastian Giacoman, Pablo J Sanchez-Millán, Jose Miguel Lozano, Miguel Álvarez-López, Laia C Belarte-Tornero, Ignasi Anguera, Benjamin Jacques Casteigt, Axel Sarrias-Mercé, Zoraida Moreno Weidmann, Concepción Alonso-Martín, Laia Llorca, Ermengol Vallés-Gras

**Affiliations:** Electrophysiology Unit, Cardiovascular Division, Hospital del Mar. Passeig Marítim de la Barceloneta, 25-29. Barcelona 08003 Spain; Electrophysiology Unit, Cardiology Department, Hospital Universitari Germans Trias i Pujol, Badalona, Spain; Electrophysiology Unit, Cardiovascular Division, Hospital del Mar. Passeig Marítim de la Barceloneta, 25-29. Barcelona 08003 Spain; Arrhythmias Unit, Heart Disease Institute, Bellvitge University Hospital, Barcelona, Spain; Department of Cardiology, Unità Operativa di Cardiologia, Azienda Ospedaliera S. Maria Nuova, Reggio Emilia 42123, Italy; Department of Cardiology, Unità Operativa di Cardiologia, Azienda Ospedaliera S. Maria Nuova, Reggio Emilia 42123, Italy; Arrhythmia Unit, Cardiology Department, Hospital Universitario Clínico San Cecilio, Granada, Spain; Arrhythmia Unit, Hospital Universitario Virgen de las Nieves, Granada, Spain; Arrhythmia Unit, Cardiology Department, Hospital Universitario Clínico San Cecilio, Granada, Spain; Arrhythmia Unit, Hospital Universitario Virgen de las Nieves, Granada, Spain; Heart Diseases Biomedical Research Group, Hospital del Mar Medical Research Institute (IMIM), Barcelona, Spain; Arrhythmias Unit, Heart Disease Institute, Bellvitge University Hospital, Barcelona, Spain; Electrophysiology Unit, Cardiovascular Division, Hospital del Mar. Passeig Marítim de la Barceloneta, 25-29. Barcelona 08003 Spain; Electrophysiology Unit, Cardiology Department, Hospital Universitari Germans Trias i Pujol, Badalona, Spain; Electrophysiology Unit, Department of Cardiology, Hospital de la Santa Creu i Sant Pau, Barcelona, Spain; Electrophysiology Unit, Department of Cardiology, Hospital de la Santa Creu i Sant Pau, Barcelona, Spain; Abbott Electrophysioloy, Chicago, IL, USA; Electrophysiology Unit, Cardiovascular Division, Hospital del Mar. Passeig Marítim de la Barceloneta, 25-29. Barcelona 08003 Spain

**Keywords:** High-density mapping, Slow pathway, Radiofrequency ablation, Conduction velocity

## Introduction

Atrioventricular nodal re-entrant tachycardia (AVNRT) is the most common re-entrant supraventricular tachycardia. The Fractionation and WaveSpeed (WS) tools, available with the Omnipolar technology (OT) and the HD-Grid mapping catheter (Abbott Medical™, Abbott Park, IL) may help in the characterization of confluent areas (CAs) of electrogram fractionation and slow conduction in the inferoseptal isthmus,^1^ in the search for safer AVNRT ablation targets.

## Methods

Non-randomized, prospective-multicentric study (NCT05531903, Ethics Committee approval no. 2022/10521). From October 2022 to August 2023, 55 consecutive AVNRT patients underwent OT-WS mapping/ablation (OT-WS group) and were compared to 64 consecutive AVNRT patients in whom a bipolar 3D map was performed (Conventional group) over the previous year.

**Figure 1:**
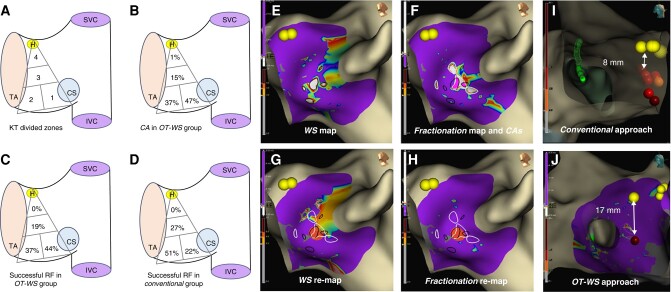
(*A*–*H*) Left lateral view of the right interatrial septum and the tricuspid valve. Panel *A* shows Koch’s triangle (KT), which was divided into four zones: Zones 1 and 2 were located at the base of the KT; Zone 1 at the septal isthmus, near the coronary sinus (CS) ostium and below its roof; and Zone 2 adjacent to Zone 1, towards the tricuspid annulus; Zone 3 and Zone 4 were located to the mid-septal and perihisian sites, respectively. Panels *B* and *C* display the percentage of confluent areas (CAs) and the percentage of successful RF applications in each zone, respectively, for the OT-WS group. Panel *D* shows the percentage of successful RF applications in each zone for the Conventional group. Panel *E* displays a WaveSpeed map, showing several zones with slow conduction located at KT’s base (slowest velocities in white, encircled in dark lines). Panel *F* shows a Fractionation Map, displaying also a few areas of fragmentation (most fragmented in white, encircled in white lines). One of these zones overlaps with two WaveSpeed slow conduction areas, delineating two small CAs (highlighted in pink colour). Panels *G* and *H* display post-ablation maps of WaveSpeed and Fractionation, respectively, showing a single (successful) RF application (dark orange dot, lesion index 4.2) in Zone 1, and the elimination of the previously encircled CAs. (*I* and *J*) Right lateral view focusing on the distance between the His signal (yellow dots) and the ablation site (red dots). A <10 mm distance is demonstrated from a patient belonging to the Conventional group (Panel *I*), while a much safer distance is noted from a patient belonging to the OT-WS group (Panel *J*). IVC, inferior vena cava; SVC, superior vena cava; H, His; TA, tricuspid annulus; CS, coronary sinus.

The initial scale for Fractionation and WS for the OT-WS group was set at ‘2’ [2–3] and ‘0.6’ [0.6–0.8], respectively.^2^ Fractionation was analysed with a 5 ms ‘width’ and 6 ms of ‘refractory’. A 3D-electroanatomical reconstruction of the Koch’s triangle (KT) was performed in sinus rhythm using the HD-Grid catheter. The KT was divided into four zones (*Figure 1*). Both Fractionation and WS maps were constructed simultaneously. The areas where high fractionation overlapped with slow conduction (CAs) were targeted for ablation. Radiofrequency (RF) lesions were considered adequate when they lasted >30 s for non-irrigated catheters (initial/maximum power 30/55 W, temperature 50–60°) or when a lesion index (LSI) > 3 was achieved for contact-force catheters. Elimination of the CA was confirmed with high-density remapping. AVNRT re-induction after elimination of the first CA lead to ablation of the following CA whenever present. Otherwise, switching to a conventional ablation was undertaken.

In the Conventional group, the ablation procedure was guided by bipolar signals from the ablation catheter obtained during KT’s 3D mapping. When an atrioventricular ratio < 1 with multicomponent and/or separate low-amplitude atrial signals was observed, RF was delivered until junctional rhythm with 1:1 retrograde ventriculoatrial conduction was elicited and maintained for 10–30 s or up to cessation of this automatic rhythm.

For both groups, ablation was considered successful if AVNRT was non-reinducible and <2 echo beats were documented.

Categorical variables were presented as percentages and compared with the χ^2^ or Fisher’s test. Continuous variables were presented as mean ± SD or median [interquartile range] as indicated. Continuous variables were compared with the Student’s *t*-test or non-parametric tests. Binomial negative and robust regression analysis were used to determine the association between the study group and the number of RF applications or the RF time adjusting for potential confounders selected by univariate analysis. The statistical analysis was performed with the SPSS 25.0 and STATA 15.1 softwares.

## Results

No baseline differences were observed between groups (*Table 1*). The number of CAs per patient was one [1–2], with 75% of patients presenting one CA, 15% two, and 5% three CAs. A majority of CAs was located in Zones 1 (47%) and 2 (37%) (*Figure 1*). The CA area was 0.51 ± 0.6 cm2. Elimination of the first CA was associated with tachycardia non-inducibility in 47 patients (86%). Ablation of the second CA in five additional patients increased the success to a 95% percentage.

**Table 1:** 

Main Results	OT-WS group *n* = 55	Conventional group *n* = 64	*P*-value
Demographics			
Age, years	54.4 ± 11.9	52 ± 16.6	0.385
Women	44 (80)	45 (70.3)	0.522
Heart disease	2 (3.6)	1 (1.6)	0.603
Redo	3 (5.5)	3 (4.7)	1
EPS parameters			
AH-interval, ms	97.8 ± 27.7	96.1 ± 23.6	0.617
HV-interval, ms	48.3 ± 7.2	47.7 ± 5.4	0.637
Proportion of patients with PR > RR interval during incremental atrial pacing	25 (45.5)	32 (50)	0.453
AH-jump	50 (91)	59 (92.2)	0.188
1:1 cycle length conduction over fast pathway (ms)	454 ± 84	463 ± 92	0580
1:1 cycle length conduction over slow pathway (ms)	355 ± 69	365 ± 78	0463
Ablation procedure (univariate analysis)			
Use of contact-force catheter	24 (43.6)	29 (45.3)	0.794
LSI in the success zone^a^	4.1 ± 1	3.5 ± 0.9	0.029
JR > 30 s	20 (36.4)	11 (17.2)	0.017
Successful RF in Zone 1	23 (44)	14 (21.8)	0.033
RF applications until success	3.9 ± 2.8	8.2 ± 6.7	<0.001
RF time until success, s	100 [63–131]	146.5 [82–226.5]	0.001
His distance (mm)	18.1 ± 6.4	15.6 ± 8	<0.06
His distance ≤ 10 mm	6 (10)	19 (29)	<0.012

Incidence rate ratio of RF applications adjusted for potential confounders (multivariable analysis)						
	Univariate			Multivariate^b^		
	Estimated coef. (CI 95%)	SE	*P*-value	Estimated coef. (CI 95%)	SE	*P*-value
OT-WS group	−35 (−59.6/−10.34)	12.4	<0.006	−40.24 (−64/−16.4)	12	<0.001
JR time > 30 s	4.9 (−23/39.8)	14.1	<0.728	5.7 (−22/33)	13.7	<0.680
Successful RF in Zone 1	14.1 (−11.2/39.4)	12.8	<0.272	21.7 (−3.8/47.2)	12.9	<0.095

Total ablation procedure time (s) adjusted for potential confounders (multivariable analysis)						
	Regressions coefficients and their significance					
	Univariate			Multivariate^c^		
	Estimated coef. (CI 95%)	SE	*P*-value	Estimated coef. (CI 95%)	SE	*P*-value
OT-WS group	−35 (−59.6/−10.34)	12.4	<0.006	−40.24 (−64/−16.4)	12	<0.001
JR time > 30 s	4.9 (−23/39.8)	14.1	<0.728	5.7 (−22/33)	13.7	<0.680
Successful RF in Zone 1	14.1 (−11.2/39.4)	12.8	<0.272	21.7 (−3.8/47.2)	12.9	<0.095

Results are expressed as mean ± standard deviation or number and (percentage).

^a^Only in patients with contact-force catheter use.

^b^Model adjusted for OT-WS group, RF application on success in Zone 1, and JR time > 30 s in at least one RF application. IRR, incidence rate ratio; CI, confidence interval; OT-WS, Omnipolar and WaveSpeed; JR, junctional rhythm; RF, radiofrequency.

^c^Model adjusted for OT-WS group, RF application on success in Zone 1, and JR time > 30 s. Coef., coefficients; CI, confidence interval; SE, standard error; OT-WS, Omnipolar and WaveSpeed; JR, junctional rhythm.

The most frequently successful ablation was Zone 1 (44%) in the OT-WS group and Zone 2 in the Conventional group (51%, *P* < 0.033; *Figure 1*). OT-WS group patients needed less RF applications (3.9 ± 2.8 vs. 8.2 ± 6.7, *P* < 0.001) and less RF time (100 [63–131] vs. 146.5 [82–227] s, *P* < 0.001) than Conventional group patients. We observed a statistical trend towards a greater distance from the ablation site to the His bundle region in the OT-WS group compared to the Conventional group (18.1 ± 6.4 mm vs. 15.6 ± 8 mm, respectively; *P* < 0.06). More importantly, a higher proportion of patients from the Conventional group showed a ≤ 10 mm distance between both signals (19/64 patients, 29%), as compared to only 6/55 patients (10%) in the OT-WS group, thus pointing towards a safer set of RF applications in the later (*P* < 0.012). In the multivariate analysis, the OT-WS-based strategy was associated with a 50% reduction in the number of RF applications (incidence rate ratio 0.534; CI 95% 0.404–0.707; *P* < 0.001) and in RF time (−40.2 s CI 95% −64/−16.4; *P* < 0.001). Although the LSI was higher in the OT-WS group, this difference was not accompanied by a reduction in the number of RF applications nor in RF time (*P* < 0.902 and *P* < 0.221, respectively; Table 1). Two patients had transient AV block in the Conventional group. The follow-up was 6 [interquartile 3–8] and 5 [interquartile 2–11] months in the OT-WS and Conventional groups, respectively (*P* < 0.7). Two patients experienced AVNRT recurrences (one per group), both being still inducible at the end of the index procedure.

## Discussion

Electrogram fragmentation demonstrated with high-density mapping (HDM) has been recently described as a useful method to localize AVNRT ablation targets.^3,4^ In this study, we provide a HDM-based technique to identify new AVNRT ablation targets (i.e. the CAs) that are noted to be modest in size and usually distant to the ‘compact’ atrioventricular node (AVN). All patients had at least one CA, and ablation using this methodology rendered the AVNRT non-inducible in 95% of cases. This strategy is associated with less RF time/applications and with a safer distance from the His region, compared to the conventional approach. Previous observations already considered of particular interest the area between the coronary sinus and the tricuspid valve, where ring tissue remnants, as continuation of inferior AVN extensions, could correspond to our CAs, most of them (84%) located at KT’s base.^1,5^ Although the use of HDM catheters increases the cost of the procedure, this could be counterbalanced in some scenarios, for instance in redo procedures. In this scenario, the increased amount of RF applications along with the eventual requirement of unusual ablation sites that could increment the risk of AV block were frequently eluded in the OT-WS group (unpublished data). Our technique might also apply to patients presenting with transient AV block during RF application due to an exceptionally low ‘fast pathway’ disposition^6^ or to some other anatomical distortions.^4^

## Limitations

This study was multicentric and non-randomized. The cumulative delivered energy was not evaluated, and a potential bias derived from this cannot be ruled out. The preset scale-bar cut-offs for the Fragmentation and WS maps are based on prior unreported experience from our group and may be considered somewhat arbitrary. The median follow-up was 6 months, and late recurrences were not registered.

## Data Availability

All relevant data are within the manuscript and its Supporting Information files.

## References

[1] Katritsis DG, Marine JE, Katritsis G, Latchamsetty R, Zografos T, Zimetbaum P, et al Spatial characterization of the tachycardia circuit of atrioventricular nodal re-entrant tachycardia. Europace 2021;23:1596–602. 34240123 10.1093/europace/euab130

[2] Pandozi C, Ficili S, Galeazzi M, Lavalle C, Russo M, Pandozi A, et al Propagation of the sinus impulse into the Koch triangle and localization, timing, and origin of the multicomponent potentials recorded in this area. Circ Arrhythm Electrophysiol 2011;4:225–34. 21372271 10.1161/CIRCEP.110.957381

[3] Wakamatsu Y, Nagashima K, Kaneko Y, Mori H, Tsutsui K, Maegaki M, et al Ablation strategy targeting the slow pathway visualized by ultrahigh-resolution mapping in typical slow-fast atrioventricular nodal reentrant tachycardia. Circ Arrhythm Electrophysiol 2023;16:e011497. 36799216 10.1161/CIRCEP.122.011497

[4] Gerontitis D, Pope MTB, Elmowafy M, Sadagopan S, Yue AM. High-density electroanatomic activation mapping to guide slow pathway modification in patients with persistent left superior vena cava. Heart Rhythm 2023;20:1018–25. 37019166 10.1016/j.hrthm.2023.03.1537

[5] Katritsis DG, Anderson RH. New insights into the mechanisms of fast and slow conduction in the atrioventricular node. Heart Rhythm 2023;20:627–30. 36049588 10.1016/j.hrthm.2022.08.025

[6] Delise P, Sitta N, Bonso A, Coro’ L, Fantinel M, Mantovan R, et al Pace mapping of Koch’s triangle reduces risk of atrioventricular block during ablation of atrioventricular nodal reentrant tachycardia. J Cardiovasc Electrophysiol 2005;16:30–5. 15673383 10.1046/j.1540-8167.2005.04054.x

